# Evaluation of humoral and cellular immune responses induced by a cocktail of recombinant African swine fever virus antigens fused with OprI in domestic pigs

**DOI:** 10.1186/s12985-023-02070-7

**Published:** 2023-05-26

**Authors:** Guanglei Zhang, Wei Liu, Sicheng Yang, Shuai Song, Yunyun Ma, Guangqing Zhou, Xiaxia Liang, Chun Miao, Junhui Li, Yanhong Liu, Junjun Shao, Huiyun Chang

**Affiliations:** 1 State Key Laboratory for Animal Disease Control and Prevention, OIE/China National Foot-and-Mouth Disease Reference Laboratory, Lanzhou Veterinary Research Institute, Chinese Academy of Agricultural Sciences, Lanzhou, Gansu Province, China; 2 Institute of Animal Health, Guangdong Academy of Agricultural Sciences, Guangzhou, China; 3 Lanzhou Institute of Biological Products Co., Ltd. (LIBP), a subsidiary company of China National Biotec Group Company Limited (CNBG), Lanzhou, 730046 China

**Keywords:** African swine fever virus, Recombinant fusion proteins, OprI, Immune responses, Vaccine

## Abstract

**Background:**

African swine fever (ASF) is a highly fatal disease in domestic pigs caused by ASF virus (ASFV), for which there is currently no commercial vaccine available. The genome of ASFV encodes more than 150 proteins, some of which have been included in subunit vaccines but only induce limited protection against ASFV challenge.

**Methods:**

To enhance immune responses induced by ASFV proteins, we expressed and purified three fusion proteins with each consisting of bacterial lipoprotein OprI, 2 different ASFV proteins/epitopes and a universal CD4^+^ T cell epitope, namely OprI-p30-modified p54-TT, OprI-p72 epitopes-truncated pE248R-TT, and OprI-truncated CD2v-truncated pEP153R-TT. The immunostimulatory activity of these recombinant proteins was first assessed on dendritic cells. Then, humoral and cellular immunity induced by these three OprI-fused proteins cocktail formulated with ISA206 adjuvant (O-Ags-T formulation) were assessed in pigs.

**Results:**

The OprI-fused proteins activated dendritic cells with elevated secretion of proinflammatory cytokines. Furthermore, the O-Ags-T formulation elicited a high level of antigen-specific IgG responses and interferon-γ-secreting CD4^+^ and CD8^+^ T cells after stimulation in vitro. Importantly, the sera and peripheral blood mononuclear cells from pigs vaccinated with the O-Ags-T formulation respectively reduced ASFV infection in vitro by 82.8% and 92.6%.

**Conclusions:**

Our results suggest that the OprI-fused proteins cocktail formulated with ISA206 adjuvant induces robust ASFV-specific humoral and cellular immune responses in pigs. Our study provides valuable information for the further development of subunit vaccines against ASF.

**Supplementary Information:**

The online version contains supplementary material available at 10.1186/s12985-023-02070-7.

## Introduction

African swine fever (ASF), is a highly contagious hemorrhagic and devastating swine viral disease that causes serious economic losses worldwide, for which there is no effective vaccine [1]. The causative agent of ASF, ASF virus (ASFV), is a large enveloped DNA virus belonging to the genus *Asfivirus* within the *Asfarviridae* family [2]. The ASFV genome varies in length between approximately 170 and 193 kbp and encodes more than 150 open reading frames [3]. Depending on the sequence of the *B646L* gene (encoding capsid protein p72), ASFV is currently classified into 24 different genotypes [4]. ASF was first reported in Kenya and has been endemic in sub-Saharan Africa for many years [5]. In 2007, it was introduced to Georgia and since then has spread to many countries of Eastern Europe [6]. In 2018, ASF emerged in China and has rapidly spread to many regions of the country and other Asian countries [7]. Millions of pigs have been killed by ASF or culled in attempts to control the disease. Currently, ASF is still prevalent in China and continues to be a serious threat to the pig industry [8]. Therefore, a safe and effective vaccine against ASF is urgently needed.

Various approaches have been evaluated for the development of vaccines against ASF [9]. However, all attempts in developing safe and effective vaccines against ASF were unsuccessful [9]. Vaccines containing inactivated viruses have not conferred protection so far [10]. Attenuated or low virulent ASFV strains have been demonstrated to elicit protective immune responses against virulent ASFV strains in swine [11–14] but, the vaccinated pigs usually experience adverse side effects, such as chronic viremia [15]. Furthermore, the application of live attenuated ASFV raises serious concerns of safety. In contrast, despite the induction of only partial protection against ASFV challenge, ASFV subunit vaccines offer a safer option [16]. A combination of recombinant p30 and p54, or a chimeric protein p54/30, protected the animals from severe disease after ASFV challenge [17, 18]. Immunization of pigs with recombinant CD2v induced the production of protective antibodies and protected 2/3 animals against ASFV challenge [19]. More recently, pEP153R has been demonstrated to be important for protection against homologous ASFV infection [20]. Interestingly, pigs immunized with a combination of recombinant p30, p72, p54 and p22 produced neutralizing antibodies but only exhibited a delayed onset of disease after challenge [21]. Growing evidence shows that cellular immune responses also play a key role in defending against ASFV infection [22, 23]. Immunization with an ASFV DNA expression library protected 60% of pigs against ASFV challenge due to antigen-specific CD8^+^ T-cells [22], revealing the existence of potential protective antigens. Together, current subunit vaccine approaches suggest the importance of selecting suitable ASFV antigens and enhancement of protective humoral and cellular immunity.

The major outer membrane lipoprotein I (OprI) of *Pseudomonas aeruginosa* is a ligand of the Toll-like receptor (TLR)-2 [24]. It is capable of triggering dendritic cells (DCs) to secrete proinflammatory cytokines in vivo, which indirectly modulates adaptive immune responses [25]. OprI acts as a natural adjuvant eliciting potent humoral and cytotoxic T cell responses against peptides/proteins when fused with it [26]. The application of OprI in fusion proteins has been extended to the antigens encoded by *B646L* and *G1340L* of ASFV, and the resulting protein was capable of inducing cytotoxic T lymphocytes [27, 28]. Different immune functions of OprI, including the promotion of Th1/Th2 responses, are attributed to activation of TLR-2 signaling [29]. The immunomodulatory activity of OprI-fusions has also been utilized in the development of vaccines [30] and recently, immunization of mice with OprI-fused antigens was shown to notably inhibit vertical transmission of *Neospora caninum* and postnatal mortality [31].

In this study, we investigated the humoral and cellular immune responses induced by a cocktail of recombinant ASFV antigens fused with OprI and their effects on ASFV infection. We designed three recombinant OprI fusion proteins and each protein contains 2 different ASFV proteins/epitopes fused with C-terminus of OprI and N-terminus of a universal CD4^+^ T cell epitope. The immunostimulatory activity of these recombinant proteins was evaluated using murine bone marrow-derived dendritic cells (BMDCs). The humoral and cellular immune responses induced by a cocktail of these three OprI fusion proteins were then assessed in pigs. The effects of immune sera and peripheral blood mononuclear cells (PBMCs) from immunized pigs on ASFV infection were determined in vitro to provide preliminary evidence before proceeding to challenge experiments in swine. We believe that our work will contribute to the development of a novel subunit vaccine against ASF.

## Materials and methods

### Cell culture and virus

Primary porcine alveolar macrophages (PAMs) were collected from Large White pigs (10–20 kg) that were negative for porcine respiratory and reproductive syndrome virus, classical swine fever virus, ASFV and pseudorabies virus. The PAMs were cultured in RPMI-1640 medium (Thermo Fisher Scientific, MA, USA) containing 15% fetal bovine serum (FBS, Thermo Fisher Scientific), at 37 °C with 5% CO_2_. ASFV China/Sichuan/2019 strains (CN/SC/19) was obtained from the Regional Laboratory of African swine fever, Lanzhou Veterinary Research Institute (LVRI, Chinese Academy of Agricultural Sciences, China). The ASFV CN/SC/19 stock used for neutralization assay was propagated and titrated in PAMs.

### Construction of fusion proteins

**Fig. 1:**
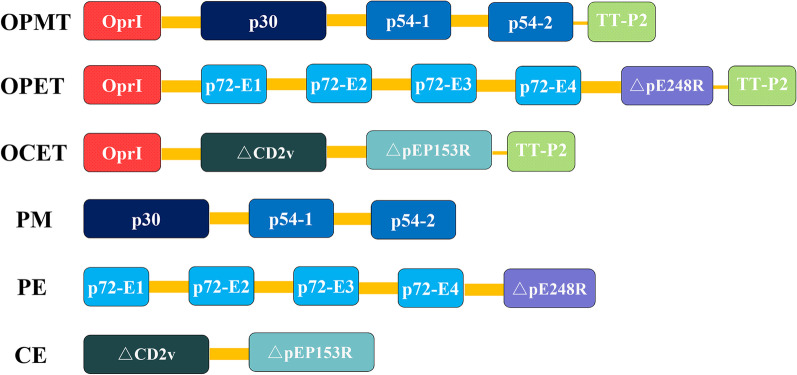
Schematic diagram of six fusion proteins, denoted as OPMT, OPET, OCET, PM, PE and CE. OprI, complete sequence of bacterial lipoprotein I; p30, complete sequence of p30; p54-1 and p54-2 respectively represents (Met1-Thr29) and (Ser53-Leu184) of p54; p72-E1, p72-E2, p72-E3 and p72-E4 respectively represents (Gln364-Pro395), (Ala518-Lys552), (Tyr179-Leu210) and (Leu242-Pro273) of p72; △pE248R, pE248R (Met1-Lys198); △CD2v, CD2v (Asp17-Tyr206); △pEP153R, pEP153R (Asn49-Lys158); TT-P2, a universal CD4^+^ T cell epitope; thick yellow line, linker 1 “ (GGGGS)_3_”; thin yellow line, linker 2 “PG”

The amino acid sequences of proteins/epitopes used to construct fusion proteins were shown in Table 1. The amino acid sequence of *P. aeruginosa* OprI was derived from the GenBank accession X13748.1 and all ASFV proteins/epitopes from the ASFV isolate China/2018/AnhuiXCGQ were retrieved from GenBank accession MK128995.1. All selected p72 epitopes, validated by experiment, were obtained from the Immune Epitope Database (IEDB [www.iedb.org]). Either extracellular or intracellular domains of pE248R, CD2v and pEP153R were selected to construct fusion proteins after protein motif analysis using SMART (http://smart.embl-heidelberg.de/). As shown in Fig. 1, three OprI-fusion proteins, including OprI-p30-modified p54-TT, OprI-p72 epitopes-△pE248R-TT and OprI-△CD2v-△pEP153R-TT were constructed and denoted as OPMT, OPET and OCET (“△” stands for truncated). In parallel, p30-modified p54, p72 epitopes-△pE248R and △CD2v-△pEP153R were designed to serve as controls and denoted as PM, PE and CE. The amino acid sequences of all fusion proteins were converted into nucleotide sequences, which were chemically synthesized (GenScript, NanJing, China) and cloned into *NdeI*-*XhoI* sites of pET30a( +) expression vector (Novagen, San Diego, CA, USA), harboring a 6 × histidine tag. The integrity and fidelity of the constructed expression plasmids were confirmed by DNA sequencing.

**Table 1:** The amino acid sequences of proteins/epitopes to construct fusion proteins

Sequence	Note	Name
MDFILNISMKMEVIF… KEVVRLMVIKLLKKK	Complete sequence of p30	p30
MDSEFFQPVY…(GGGGS)_3_… YTHKDLENSL	p54 (His30-Phe52) replaced by (GGGGS)_3_	Modified p54
QKDLVNEFPGLFVRQSRFIAGRPSRRNIRFKP	p72 (Gln364-Pro395)	p72 epitope 1
ACSSISDISPVTYPITLPIIKNISVTAHGINLIDK	p72 (Ala518-Lys552)	p72 epitope 2
YCEYPGERLYENVRFDVNGNSLDEYSSDVTTL	p72 (Tyr179-Leu210)	p72 epitope 3
LCNIHDLHKPHQSKPILTDENDTQRTCSHTNP	p72 (Leu242-Pro273)	p72 epitope 4
MGGSTSKNSFKNTT… NFFDFIADAISAVFK	pE248R (Met1-Lys198)	△pE248R
DYWVSFNKTIILDSN… LTLSSNYFYTFFKLY	CD2v (Asp17-Tyr206)	△CD2v
NKPICYQNDDKIFYC… YKKQKHVSLLYICSK	pEP153R (Asn49-Lys 158)	△pEP153R
MNNVLKFSALALAAV… NERALRMLEKASRK	Complete sequense of OprI	OprI
QYIKANSKFIGITELLE	tetanus toxoid CD4^+^ T cell epitope P2	TT-P2

### Expression and purification of recombinant fusion proteins

Each expression plasmid was transformed into competent *Escherichia coli* BL21 (DE3) pLysS cells (Takara, Dalian, China), and the resulting positive strains were induced with 1 mM isopropylthio-β-galactoside at an optical density (OD) of 0.6 at 600 nm for expression of the fusion proteins. OPMT, OPET and OCET were isolated from the bacterial outer membranes as previously described [32]. Briefly, the bacterial pellets were resuspended in Tris–EDTA buffer (pH 8.0) containning 2.5 mg/mL lysozyme (Genscript) and incubated for 35 min on ice. Following addition of an equal volume of sarkosyl (2%, w/v), the mixture was ultra-sonicated and then ultra-centrifuged at 100,000 × g for 2 h at 4 °C to obtain insoluble outer membrane pellets. After being treated with chloroform/methanol (2:1, v/v) to extract redundant lipids, the outer membranes were solubilized in 6 M guanidine hydrochloride and subsequently purified by Ni-affinity chromatography in the presence of 8 M urea. By contrast, PM, PE and CE were purified from the inclusion bodies of their induced bacteria under the same chromatographic process. All eluted recombinant fusion proteins were refolded by dialysis. Endotoxin was then removed by phase separation using Triton X-114. The endotoxin level in the purified protein samples was determined with a ToxinSensor™ Chromogenic LAL Endotoxin Assay Kit (Genscript).

### SDS-PAGE and western blot analysis

Approximately equal amounts of each recombinant protein were subjected to 12% SDS-PAGE. The gel was stained with Coomassie blue or transferred to polyvinylidene difluoride membranes (Milipore, San Diego, CA, USA). The membranes were blocked in 5% skim milk for 2 h at room temperature (RT) followed by incubation with anti-histidine monoclonal antibody (mAb) (1:5000 dilution, Abcam, Cambridge, UK) or anti-ASFV swine serum (1:300 dilution, China Institute of Veterinary Drug Control, Beijing, China) overnight at 4 °C. After washing five times with 0.05% Tween-20 in PBS (PBST), the membranes were incubated with horseradish peroxidase (HRP)-conjugated goat anti-mouse IgG (1:5000 dilution, Abcam) or HRP-conjugated goat anti-pig IgG (1:5000 dilution, Abcam) for 1 h at RT. After washing with PBST, the blots were developed with enhanced chemiluminescence reagent (Thermo Fisher Scientific).

### Dendritic cell stimulation

All recombinant fusion proteins were assessed for DCs stimulation. BMDCs were generated as previously described [33]. The BMDCs were collected and adjusted to a density of 5 × 10^5^ cells/mL, and further cultured in the presence of each protein (1 or 5 µg/ml), lipopolysaccharide (LPS, 0.1 µg/ml) or medium for 24 h. Tumor necrosis factor-α (TNF-α) and interleukin-12p70 (IL-12p70) were measured in the culture supernatants by commercial ELISA kits (Neobioscience, ShenZhen, China).

### Vaccine preparation and immunization

Two groups of antigens, one consisting of OPMT (150 μg/mL), OPET (150 μg/mL) and OCET (300 μg/mL) and the other of PM (150 μg/mL), PE (150 μg/mL) and CE (300 μg/mL), were respectively emulsified with an equal volume of Montanide ISA206 (ISA206, SEPPIC Inc., Paris, France) to form a water-in-oil-in-water (W/O/W) blends, namely O-Ags-T formulation (OPMT + OPET + OCET + ISA206) and Ags formulation (PM + PE + CE + ISA206). A total of 11 six-week-old, healthy outbred pigs were obtained from a local farm and group-housed in the Large Animal Research Center of LVRI. The animal care protocols were approved by the Institutional Animal Care and Use Committee (IACUC) of LVRI. All pigs were randomly divided into three groups (n = 4 for group 1 and group 2, n = 3 for the control group) and were immunized twice via intramuscular route at 3-week intervals. Animals in group 1 and group 2 were immunized with O-Ags-T formulation and Ags formulation (2 mL/pig), respectively. Control pigs were given an equal volume of PBS formulated with ISA206. All pigs were bled at 0, 14, 21, 35, and 42 days post vaccination (dpv).

### Detection of antigen-specific IgG

Antigen-specific IgG responses in the sera from vaccinated pigs were measured by indirect ELISA. Briefly, 96-microwell plates (Costar, Cambridge, MA, USA) were pre-coated with recombinant p30 (0.125 µg/mL), modified p54 (0.5 µg/mL), p72 (1 µg/mL), △pE248R (1 µg/mL), △CD2v (2 µg/mL) and △pEP153R (2 µg/mL) overnight at 4 °C and blocked with 5% skim milk for 2 h at 37 °C. Serum samples (1:100 dilutions) from immunized pigs were added to each well and incubated for 1 h at 37 °C. In parallel, blank and negative controls were set up. After five washes with PBST, the plates were incubated with anti-pig IgG-HRP (1:10,000 dilutions) for 1 h at 37 °C. After a final wash series, color were developed with 3,3’,5,5’-tetramethylbenzidine substrate for 15 min at 37 °C and stopped by 2 M H_2_SO_4_. The OD at 450 nm (OD_450_) of each well was determined using a microplate reader (Thermo Fisher Scientific).

### Assessment of lymphocyte proliferation

Peripheral blood samples were collected from each immunized pig at 42 dpv into sodium heparin vacutainer tubes. PBMCs were isolated from heparinized blood using lymphocyte separation solution (Dakewei, Shenzhen, China) and stained with 5(6)-carboxyfluorescein diacetate succinimidyl ester (CFSE, Invitrogen, San Diego, CA, USA) for the detection of cell proliferation. Briefly, A total of 2 × 10^6^ PBMCs were incubated with 1 mL of CFSE labeling solution (1.25 µM) for 10 min at 37 °C. After washing three times with PBS, the cells were resuspended in RPMI-1640 containing 5% FBS at 1 × 10^6^ cells/mL and cultured in 24-well plates (1 × 10^6^ cells /well, Corning, NY, USA) in the presence of heat-inactivated ASFV (10^5^ 50% hemadsorbing doses (HAD_50_)) for 72 h. In parallel, unstained cells and stained cells treated with concanavalin A (5 μg/mL, Dakewei) or medium were established as blank, positive and negative controls, respectively. The cells were collected for proliferation analysis by flow cytometry, indicated by decreased CFSE fluorescence intensity (CFSE-low).

### Determination of IFN-γ-producing T lymphocyte by intracellular cytokine staining

PBMCs were obtained as described above and seeded into 24-well plates (1 × 10^6^ cells 1 mL/well). After addition of inactivated ASFV (10^5^ HAD_50_) to each well, the cells were cultured for 40 h and then incubated with monensin (1.7 μg/mL, Biolegend, San Diego, CA, USA) for a further 8 h. In parallel, PBMCs incubated with a cell activation cocktail (1:1000 dilution, Biolegend) or medium were established as positive and negative controls. The cells of each well were collected and stained with PerCP/Cyanine dye 5.5-conjugated anti-CD3, fluorescein isothiocyanate-conjugated anti-CD4 and phycoerythrin-conjugated anti-CD8α mAbs (all from BD Biosciences, San Diego, USA) for 30 min at 4 °C. After treating with Fix and Perm reagents (BD Biosciences), the cells were stained with Alexa Fluor 647 (AF647)-conjugated anti-IFN-γ mAb (BD Biosciences) for 30 min. The cells were washed with PBS and subjected to flow cytometry analysis. The cells were gated to select CD3^+^ T lymphocytes, within which the gate for CD4^+^ and CD8^+^ T cells were further determined. The percentages of IFN-γ-producing (IFN-γ^+^) CD4^+^ and CD8^+^ T cells were analyzed for each sample.

### Neutralization assay

The neutralization activity of sera from each group collected at 0 (pre-immune sera) and 42 dpv (immune sera) was assessed as previously described [34]. ASFV CN/SC/19 (multiplicity of infection (MOI) = 0.01, calculated by PAMs for inoculation) was mixed with 200 μL of heat-inactivated sera (dilution: 1/5) or equal volume of medium (negative control) and incubated overnight at 37 °C. The sera/ASFV mixture were inoculated onto PAMs monolayers in 24-well plates. After virus adsorption for 1 h at 37 °C, the viral inocula were removed and RPMI-1640 with 5% FBS was added (500 μL/well) with a further incubation for 48 h. For each sample, three replicates were included. The ASFV genome copy number in each sample was determined by quantitative polymerase chain reaction (qPCR) kits for ASFV (Diagnostic Products Center, LVRI). The percentage of neutralization for each serum sample was computed as follows:

Neutralization (%) = 100 – 100 × copy number of ASFV in the presence of immune serum/copy number of ASFV in the presence of pre-immune serum.

The neutralization activity of the immune sera at 42 dpv was also evaluated by indirect immunofluorescence analysis. The sera were incubated with ASFV and inoculated on PAMs monolayers as described above. After incubation for 48 h, the cells were washed with PBS and fixed with 4% paraformaldehyde for 1 h at 4 °C. After permeabilization with 0.25% Triton X-100 for 10 min, the cells were blocked with 5% BSA for 1 h and further incubated with anti-p30 mAb (1:300 dilutions, produced by our laboratory) for 1 h. After washing with PBS, the cells were incubated with tetraethyl rhodamine isothiocyanate (TRITC)-conjugated goat anti-mouse IgG (1:500 dilutions, Abcam) for 1 h followed by staining with DAPI (Thermo Fisher Scientific) for 5 min. After three washes with PBS, images recording were performed with a fluorescence microscope (Leica, Wetzlar, Germany). 

### Assessment of ASFV infection in PBMCs

PBMCs from immunized pigs at 42 dpv were obtained as described above and cultured in 24-well plates (500 μL/well) at 1 × 10^6^ cells/mL and infected with ASFV CN/SC/19 (MOI = 0.01) for 48 h at 37 °C. PBMCs from non-immunized healthy pigs inoculated with equal amounts of ASFV serve as a control. For each sample, three replicates were conducted. The ASFV genome copy number in each sample was determined by qPCR kits for ASFV, comparing them with those that appeared in PBMCs from non-immunized pigs. The inhibition percentage of ASFV infection for each group of PBMCs was calculated as follows:

Inhibition (%) = 100 – 100 × copy number of ASFV in PBMCs from immunized pigs/copy number of ASFV in PBMCs from nonimmunized pigs.

### Statistical analyses

All experiments were repeated three times with consistent results. Data in figures are expressed as mean ± standard deviation (SD), as indicated in figure legends. The statistical significance of differences between groups was determined by one-way analysis of variance and Student’s t-tests (GraphPad Prism software, San Diego, CA, USA). No significance (NS) between groups was established at *P* > 0.05. * P < 0.05, ** P < 0.01 and *** P < 0.001 were considered to be statistically significant differences between groups.

## Results

### Purification and identification of recombinant proteins

**Fig. 2:**
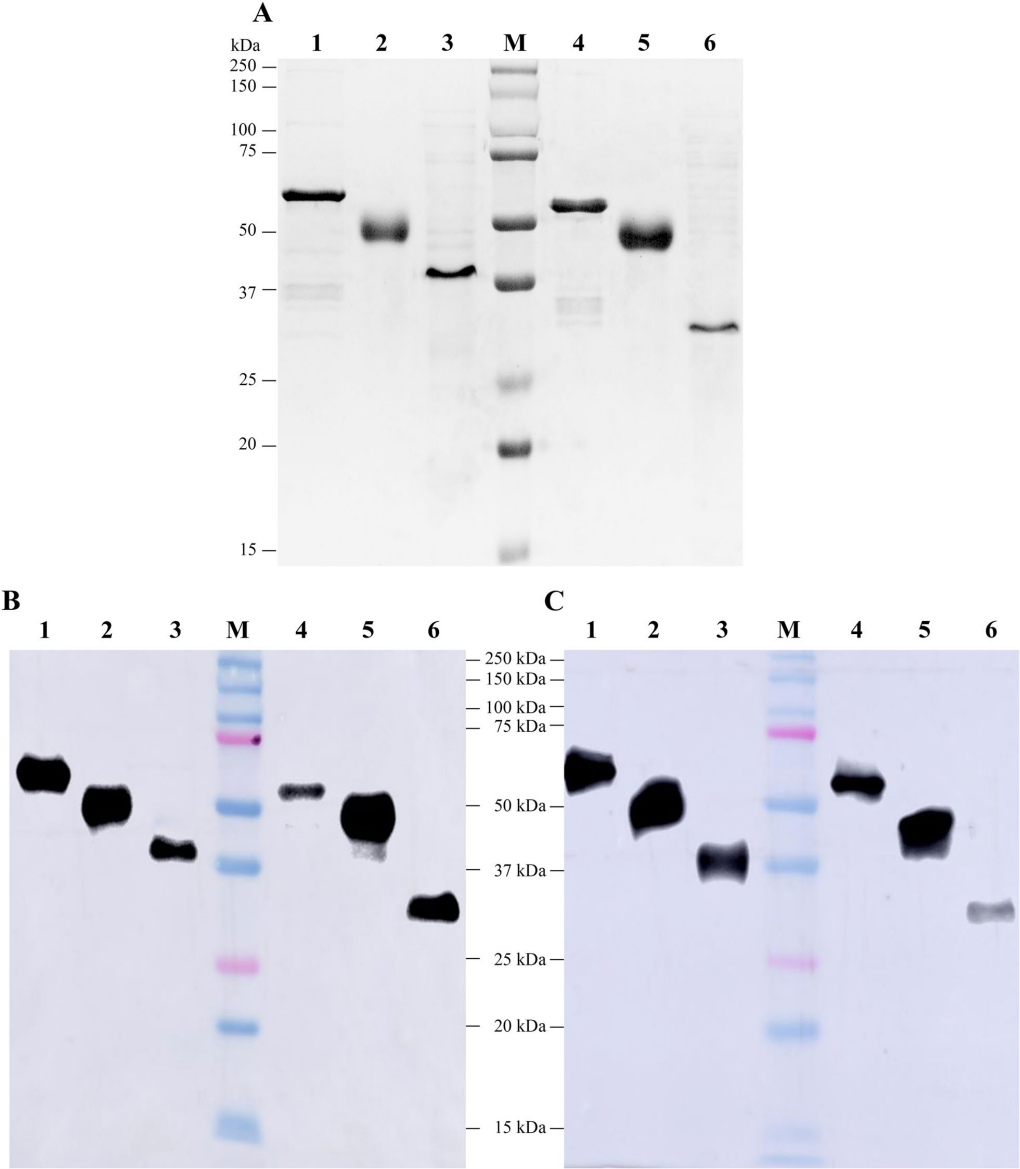
Identification of purified recombinant fusion proteins by SDS-PAGE and western blotting. **A** SDS-PAGE analysis of purified recombinant proteins. Lane M, molecular weight markers; lane 1, OPMT; lane 2, OPET; lane 3, OCET; lane 4, PM; lane 5, PE; lane 6, CE. Western blotting confirmation of purified recombinant proteins with anti-polyhistidine mAb (**B**) or anti-ASFV swine serum (**C**) as primary antibody and corresponding secondary antibodies. Lane contents are the same as in panel (**A**)

The expression vector containing the genes encoding each fusion protein was successfully constructed and transformed in *E. coli* for protein expression. These His-tagged recombinant proteins were purified by Ni-affinity chromatography and verified by SDS-PAGE (Fig. 2A). Bands corresponding to OPMT, OPET, OCET, PM, PE and CE were clearly visible after staining with Coomassie blue. The purified recombinant proteins were also confirmed by western blot, using an anti-histidine mAb (Fig. 2B) and anti-ASFV swine serum (Fig. 2C). After endotoxin removal, the concentration of endotoxin in the purified recombinant proteins was detected by Limulus amebocyte lysate assays and shown to be below 0.1 EU/µg.

### Increased stimulation of dendritic cells by OprI-fusion proteins

**Fig. 3:**
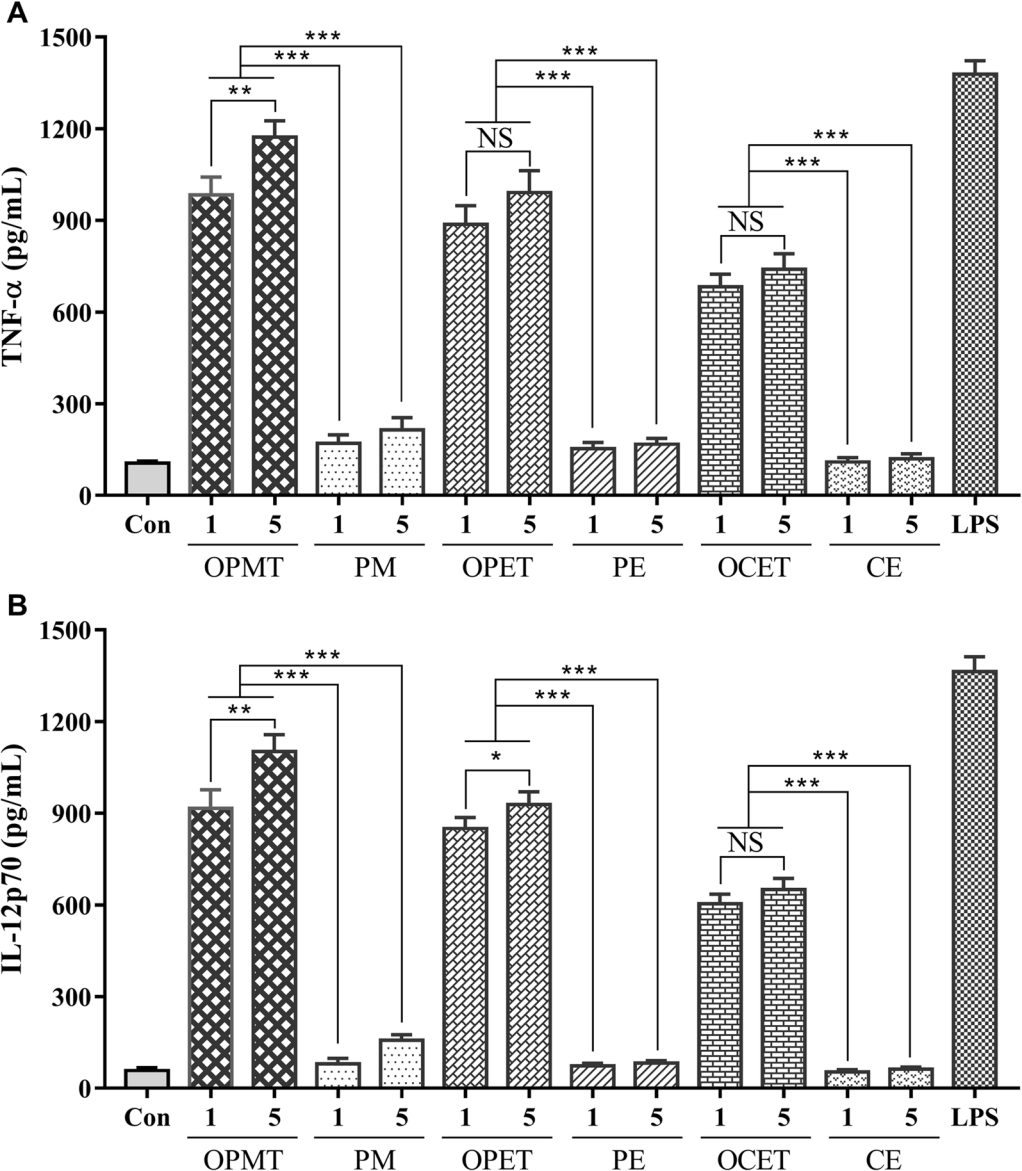
Cytokines secreted by DCs after stimulation. TNF-α (**A**) and IL-12p70 (**B**) levels in culture supernatants following stimulation of BMDCs with each recombinant protein (1 or 5 μg/mL), LPS (0.1 µg/mL) or medium (control) for 24 h. All data are displayed as mean ± SD (n = 3); NS = *P* > 0.05; *P < 0.05, **P < 0.01, ***P < 0.001

The immunostimulatory activity of the recombinant proteins was evaluated on BMDCs. The levels of TNF-α (Fig. 3A) and IL-12p70 (Fig. 3B) in the culture supernatants of BMDCs incubated with 1 μg/mL or 5 μg/mL of OPMT, OPET and OCET were significantly higher than that of PM, PE and CE. Amongst all the recombinant proteins, OPMT induced the highest levels of TNF-α and IL-12p70 at either concentration. These results suggest that OprI-fused proteins are able to induce strong and specific stimulation of DCs.

### OprI-fusion proteins cocktail elicited high levels of ASFV antigen-specific IgG responses in pigs

**Fig. 4:**
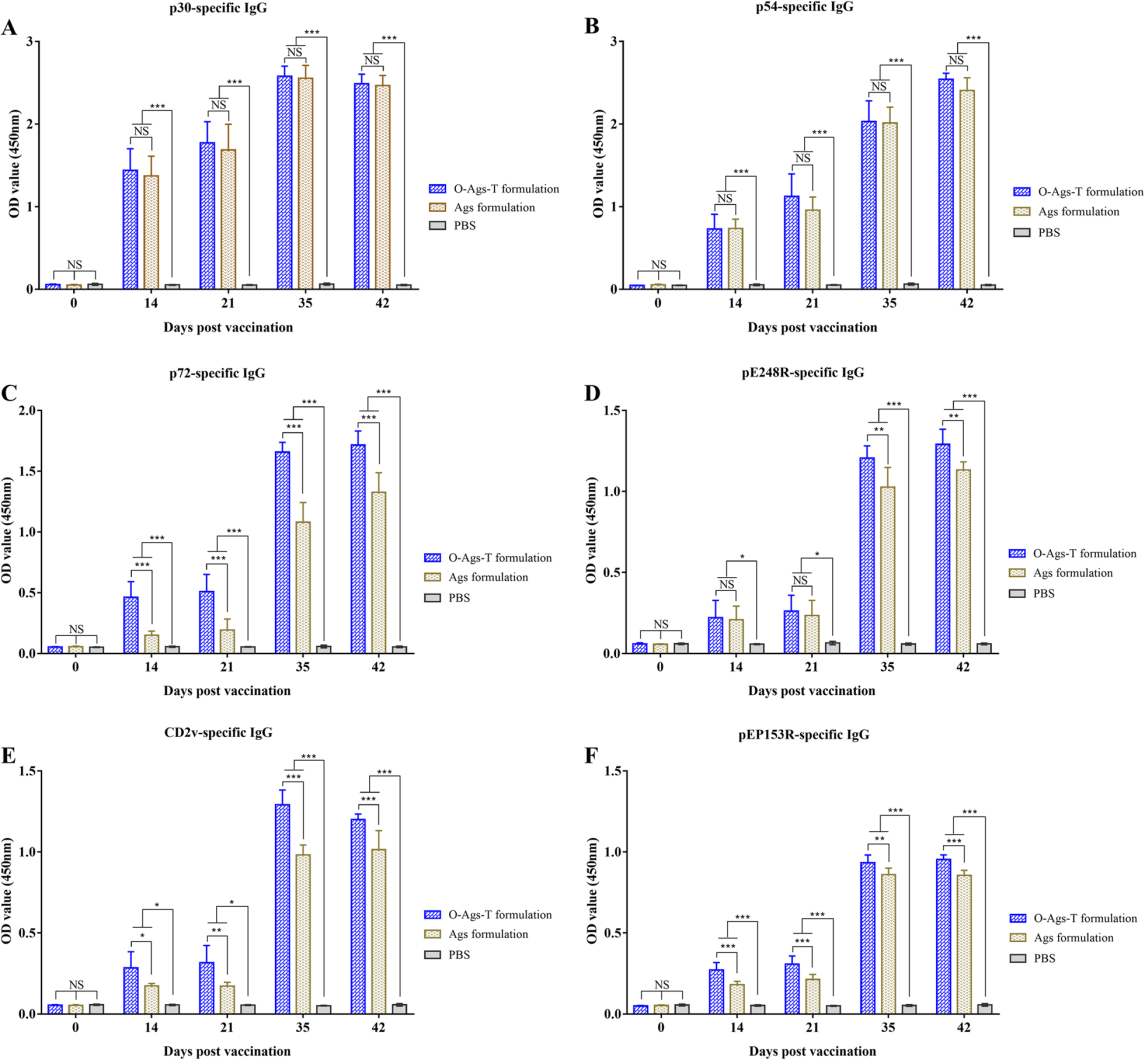
Antigen-specific IgG detection. IgG responses to p30 (**A**), p54 (**B**), p72 (**C**), pE248R (**D**), CD2v (**E**) and pEP153R (**F**) in the sera from each immunized pig at 0, 14, 21, 35 and 42 dpv were detected by indirect ELISAs, with serum dilutions of 1:100 and goat anti-pig IgG-HRP dilutions of 1:10,000. The results are expressed as OD_450_ (mean ± SD). NS = *P* > 0.05; *P < 0.05; **P < 0.01; ***P < 0.001

IgG responses in immunized pigs were measured by indirect ELISA. As shown in Fig. 4A–E, The level of p30-, p54-, p72-, pE248R-, CD2v-, and pEP153R-specific IgG were detectable at 14 dpv in the serum from pigs that received O-Ags-T formulation or Ags formulation and increased to a higher level after a booster vaccination (at 35 and 42 dpv). By contrast, all six kinds of antigen-specific IgGs were barely detectable in the sera from all pigs at 0 dpv and from pigs immunized with PBS at any time point. Comparing with pigs that received Ags formulation, pigs immunized with O-Ags-T formulation produced similar levels of p30- and p54-specific IgGs, with no significant difference between them at the same time point (*P* > 0.05). However, immunization of pigs with O-Ags-T formulation induced significantly higher levels of p72-, pE248R-, CD2v- and pEP153R-specific IgGs at 35 and 42 dpv, relative to immunization with Ags formulation. Taken together, the data indicates that immunization with a cocktail of OprI-fusion proteins formulated with ISA206 efficiently elicited ASFV antigen-specific IgG responses in pigs.

### Immunization with OprI-fusion proteins cocktail enhanced ASFV-specific cellular immune responses

**Fig. 5:**
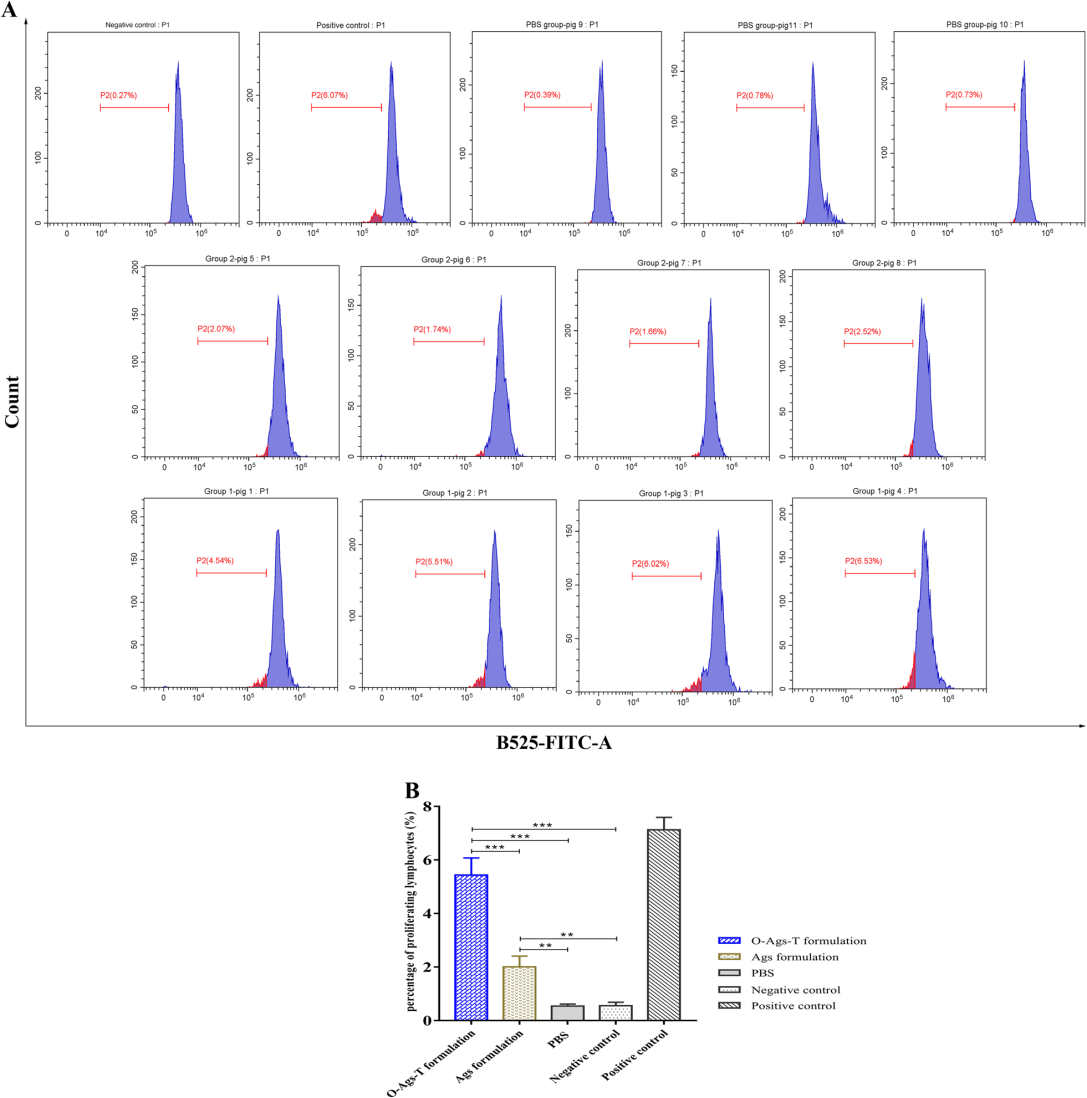
Lymphocyte proliferation in PBMCs from immunized pigs after in vitro stimulation with inactivated ASFV. **A** The percentage of CFSE-low lymphocytes (represented by P2) detected by flow cytometry in PBMCs from immunized pigs at 42 dpv after staining with CFSE and incubation with inactivated ASFV, medium (negative control) or concanavalin A (positive control). Group 1 and group 2 respectively represent pigs immunized with O-Ags-T formulation or Ags formulation. **B** Calculated percentages of proliferative lymphocytes based on CFSE-low lymphocytes in PBMCs from three separate experiments. The graphs show mean results with error bars indicating the SD; **P < 0.01; ***P < 0.001

To determine cellular immune responses in immunized pigs, proliferation of lymphocytes in PBMCs from each pig at 42 dpv were assessed by CFSE labeling. As shown in Fig. 5A, pigs that received O-Ags-T formulation exhibited the highest percentage of CFSE-low lymphocytes among three groups when stimulated with inactivated ASFV, which indicated the highest proliferation of lymphocytes. Pigs immunized with Ags formulation showed a moderated proliferation of lymphocytes after stimulation. In contrast, proliferation of lymphocytes was not detected in PBMCs from PBS-immunized pigs. The percentages of proliferative lymphocytes were determined in three independent experiments (Fig. 5B). Immunization of pigs with O-Ags-T formulation induced significantly higher percentage of lymphocyte proliferation than immunization with Ags formulation or PBS (*P* < 0.001).

**Fig. 6:**
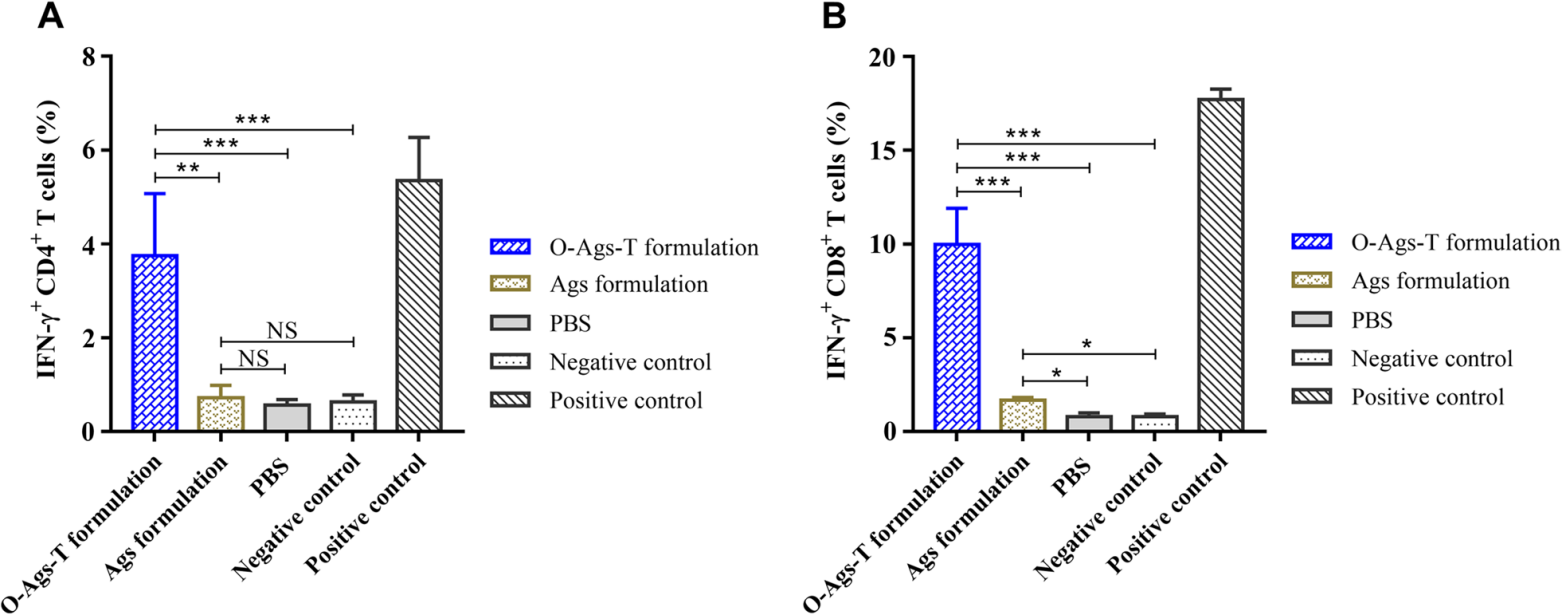
IFN-γ-producing T cells in PBMCs from immunized pigs at 42 dpv. The percentage of IFN-γ-secreting CD4^+^ T cells (**A**) and IFN-γ-secreting CD8^+^ T cells (**B**) in PBMCs from immunized pigs at 42 dpv after in vitro stimulation with inactivated ASFV, medium (negative control) or a cell activation cocktail (positive control). The graphs show mean results with error bars indicating the SD. NS = *P* > 0.05; *P < 0.05; **P < 0.01; ***P < 0.001

To further investigate the types of reactive lymphocytes, the percentage of IFN-γ^+^ CD4^+^ and CD8^+^ T cells in PBMCs from immunized pigs at 42 dpv were determined by flow cytometry. The CD3^+^, CD4^+^ and CD8^+^ T cells were gated one by one (Additional file 1: Fig. S1). After stimulation with inactivated ASFV, PBMCs from all pigs immunized with O-Ags-T formulation showed high percentages of IFN-γ^+^ CD4^+^ and IFN-γ^+^ CD8^+^ T cells (Additional files 2 and 3: Figs. S2 and S3). The percentages of IFN-γ^+^ CD4^+^ and IFN-γ^+^ CD8^+^ T cells in each group were determined in three independent experiments (Fig. 6A and B). The percentages of both IFN-γ^+^ CD4^+^ and IFN-γ^+^ CD8^+^ T cells from pigs that received O-Ags-T formulation were significantly higher than the other groups, indicating robust T cell mediated immune responses. The average percentage of IFN-γ^+^ CD8^+^ T cells from pigs that received O-Ags-T formulation was about two times higher than that of IFN-γ^+^ CD4^+^ T cells, indicating stronger ASFV-specific CD8^+^ T cell responses. These data demonstrate that immunization of pigs with OprI-fusion proteins cocktail elicited robust ASFV-specific cellular immune responses, which may contribute to defend against ASFV infection.

### Sera from immunized pigs neutralized ASFV infection in vitro

**Fig. 7:**
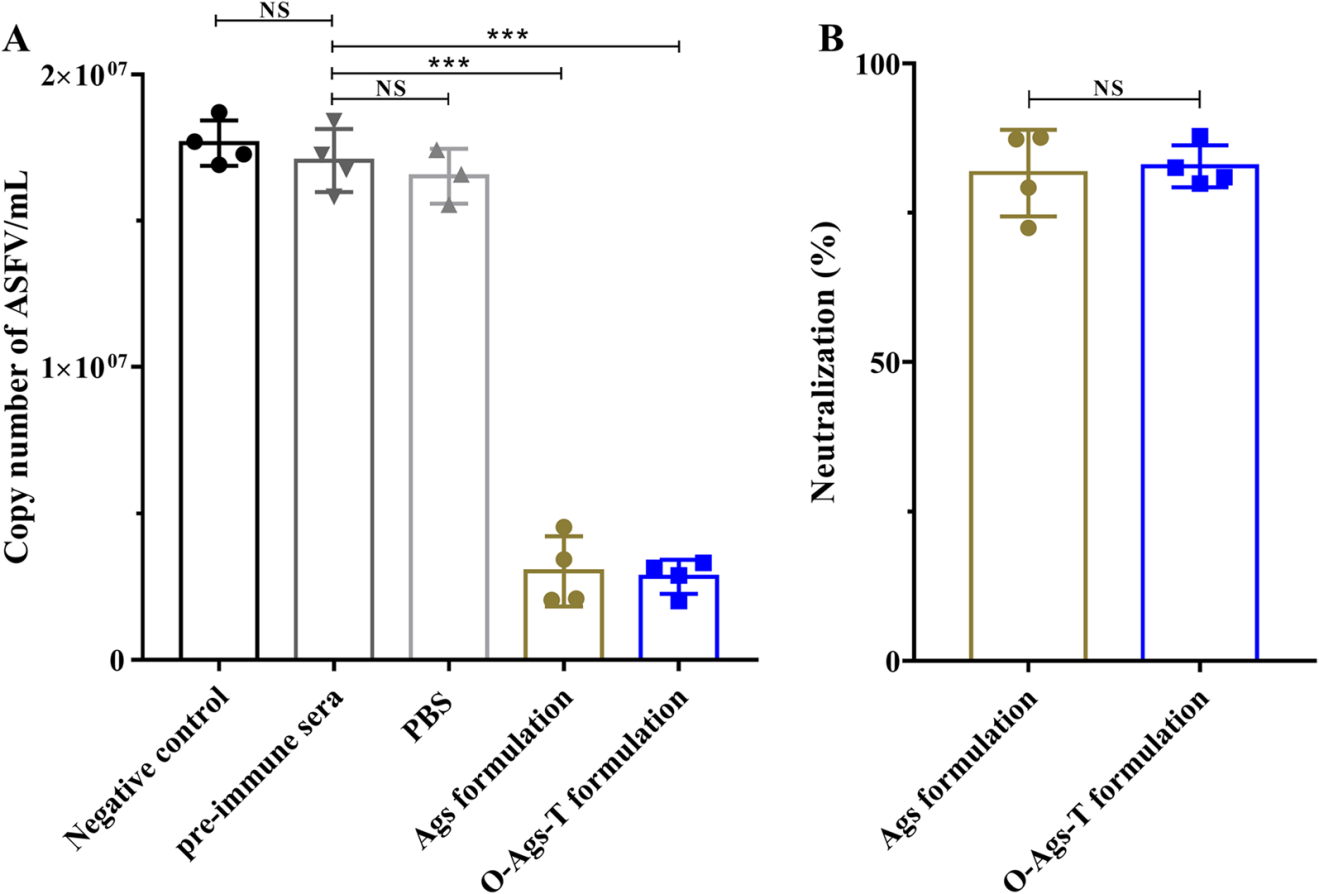
Neutralization of ASFV infection by the sera from immunized pigs at 42 dpv. **A** The ASFV genome copy number in PAMs infected by ASFV (MOI = 0.01) preincubated with heat-inactivated pre-immune sera, immune sera (both at 1:5 dilution) or equal volume of medium (negative control), respectively. **B** The percentage of ASFV neutralization by the sera from pigs immunized with O-Ags-T formulation or Ags formulation at 42 dpv. The mean results with standard error are shown. NS = *P* > 0.05; ***P < 0.001

To analyze ASFV neutralizing activity of the immune sera, their effect on ASFV proliferation in PAMs monolayers was determined. In the first approach, the ASFV genome copy number in each sample were determined by qPCR. As shown in Fig. 7A, compared with the control group, the sera from pigs immunized with either O-Ags-T formulation or Ags formulation significantly decreased ASFV genome copy number (*P* < 0.001), indicating inhibition of ASFV proliferation in PAMs. In contrast, the sera from PBS-immunized pigs and all pigs prior to immunization exhibited no ability to reduce the copy number of ASFV genome. Based on the results, the percentages of ASFV neutralized by the sera from pigs immunized with O-Ags-T formulation or Ags formulation were calculated. The average percentages of virus neutralized by the sera from pigs that received O-Ags-T formulation or Ags formulation were 81.6% and 82.8% respectively, with no significant difference between them (*P* > 0.05) (Fig. 7B).

**Fig. 8:**
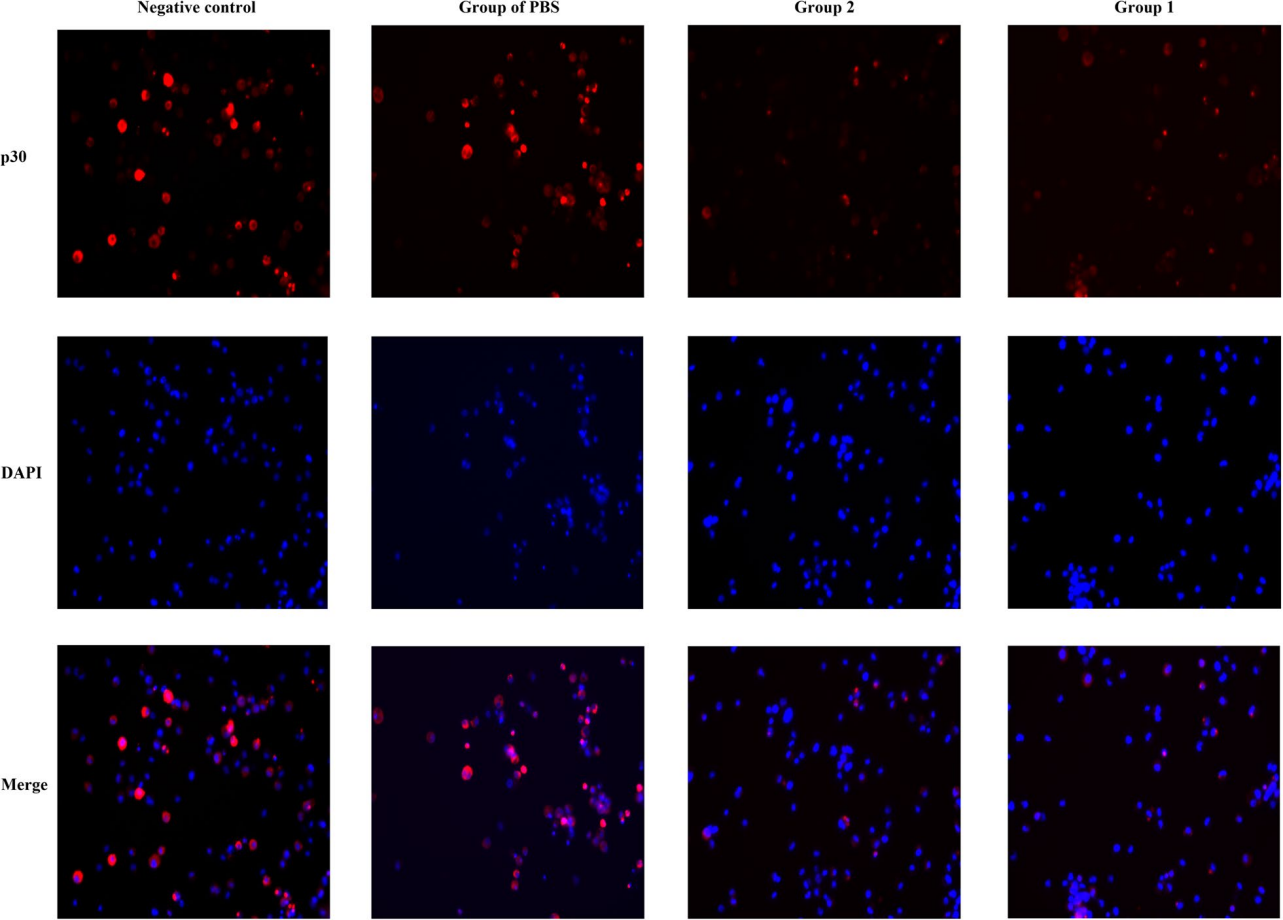
Indirect immunofluorescence identification of PAMs infected with ASFV. PAMs infected with ASFV (MOI = 0.01) preincubated with heat-inactivated immune sera (42 dpv) from group 1 (O-Ags-T formulation), group 2 (Ags formulation) and PBS group or medium (negative control) were subjected to indirect immunofluorescence assay, with anti-p30 mAb as primary antibody and goat anti-mouse IgG-TRITC as secondary antibody. DAPI indicates the staining of the nucleus. The lower panel represents the merge of the 2 channels above

The ASFV neutralizing activity of immune sera was also determined from the reduced presence of ASFV in PAMs by indirect immunofluorescence. In contrast to ASFV without sera incubation (negative control), incubation of ASFV with the sera from pigs immunized with O-Ags-T formulation or Ags formulation was shown to remarkably reduce TRITC intensity and the number of TRITC positive cells after infection (Fig. 8). By contrast, there was no visible alteration in PAMs infected with ASFV preincubated with PBS-immunized sera. These results confirmed the antibodies induced by O-Ags-T formulation or Ags formulation in pigs were capable of neutralizing ASFV infectivity in vitro.

### PBMCs from immunized pigs inhibit ASFV infection in vitro

**Fig. 9:**
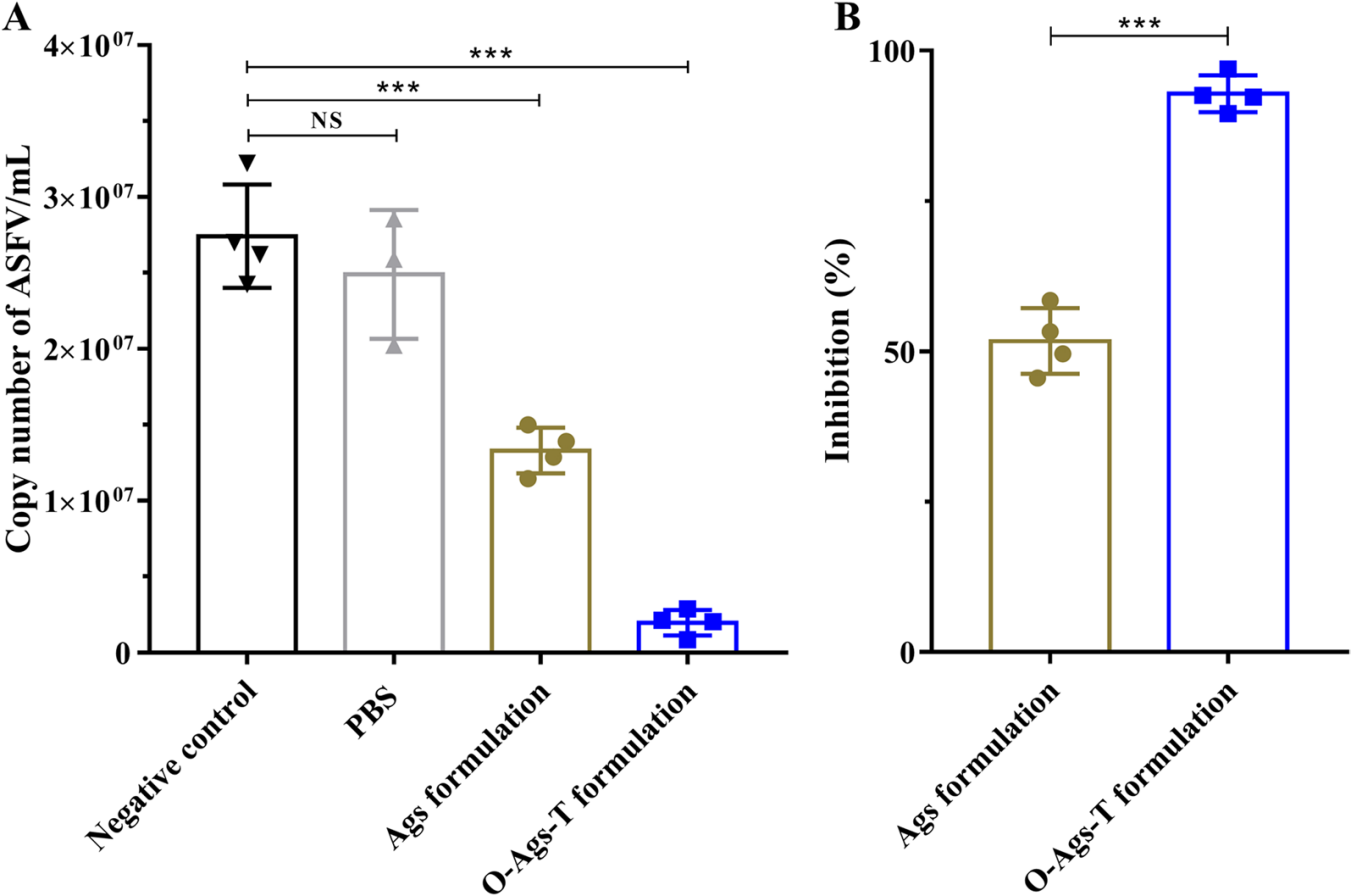
Inhibition of ASFV infection in PBMCs from immunized pigs at 42 dpv. **A** Copy number of ASFV genome in PBMCs from non-immunized pigs or immunized pigs at 42 dpv infected with ASFV (MOI = 0.01). **B** ASFV inhibition percentages in PBMCs from pigs immunized with O-Ags-T formulation or Ags formulation at 42 dpv. The mean results with standard error are shown. NS = *P* > 0.05; ***P < 0.001

PBMCs isolated from immunized pigs at 42 dpv were infected with ASFV to investigate their effects on ASFV infection. PBMCs from non-immunized or PBS-immunized pigs both showed a higher ASFV genome copy number after infection, with no significant difference between them (*P* > 0.05) (Fig. 9A). In contrast to PBMCs from non-immunized pigs, the copy number of ASFV genome significantly decreased in PBMCs from pigs immunized with O-Ags-T formulation or Ags formulation (*P* < 0.001), indicating their inhibitory effects on ASFV infection in vitro. On the basis of above results, the percentage inhibition of ASFV infection in PBMCs from these two groups were calculated and shown in Fig. 9B. PBMCs from pigs immunized with O-Ags-T formulation inhibited ASFV infection in vitro by as much as 92.6%, which was significantly higher than that of pigs immunized with Ags formulation (*p* < 0.001). This suggests an enhancement of the antiviral effect of cellular immune responses after pigs immunization with the OprI-fused proteins cocktail.

## Discussion

The development of safe and effective vaccines against ASF is very challenging due to the complex nature of the virus [8]. Amongst the approaches to develop ASF vaccines, live attenuated ASFV strains generated by targeted gene deletion appear to be very promising due to their induction of robust protective immunity against ASFV challenge, but this approach still suffers from the risk of a reversion to virulence [35]. In contrast, subunit-based vaccines against ASFV avoid these potential problems and seem to be easier to manufacture than attenuated ASFV [36]. Different kinds of subunit-based vaccines against ASF have been evaluated in pigs with ASFV challenge and some of them conferred a certain degree of protection [17–19, 36], while a few of them provided no protection, even though more ASFV antigens were included in the vaccines [21, 37]. These results highlight the importance of selecting suitable ASFV antigens and enhancement of protective humoral and cellular immunity in the development of subunit vaccines against ASF.

In the present study, three recombinant fusion proteins based on p30, p54, p72, pE248R, CD2v and pEP153R were constructed and used to prepare a cocktail vaccine against ASF. Amongst the selected ASFV proteins, p30, p54, p72 and CD2v have been extensively studied in subunit vaccines and shown to induce protective immune responses or neutralizing antibodies against ASFV. The other two ASFV proteins, pE248R and pEP153R, were demonstrated to be potential protective antigens in genetic knockout experiments [20, 38]. Due to the difficulty in expression of membrane proteins and proteins more than 100 kDa in *E. coli* [39], all amino acid sequences of membrane proteins (p54, pE248R, CD2v, pEP153R) were modified or truncated according to protein motif analysis by SMART and the identified p72 epitopes were used instead of the complete sequence of p72. To enhance humoral and cellular immune responses induced by ASFV proteins, OprI and the universal CD4^+^ T cell epitope P2 (aa 830–844) of tetanus toxoid (TT-P2) were included to construct each recombinant fusion protein. On the one hand, OprI, targeting TLR-2, can be efficiently taken up by monocytes/macrophages and improve antigen-specific immune responses [26]. On the other hand, TT-P2 has been shown to enhance the immunogenicity of a peptide vaccine for malaria [40] and an epitope-based vaccine for rotavirus [41]. The main purpose of our study is to enhance immune responses induced by ASFV proteins and further evaluate the vaccine potential of a cocktail of three OprI-fused proteins.

Since DCs are professional sentinels in the immune system and vital for the induction of immune responses [42], murine BMDCs were used to assess the stimulatory activity of the recombinant proteins. The OprI-fused proteins were able to activate DCs, as demonstrated by secreting higher levels of TNF-α and IL-12p70. The levels of TNF-α induced by OPMT and OPET were similar to OprI-fusion proteins in a previous study [31], while the level of TNF-α induced by OCET was relatively lower. The most likely reason for this is the presence of inhibitory motifs in CD2v or pEP153R. This deduction was confirmed by a recent study, where pEP153R was found to decrease the expression of MHC class I [43]. The maturation and activation of DCs triggered by OprI-fused proteins were attributed to activate the TLR2/4 signaling pathway [25]. These results suggest that OprI-fused proteins were capable of activating DCs, and may contribute to the activation of T-cell mediated immune responses.

The data from ELISA showed that immunization with O-Ags-T formulation or Ags formulation induced varied levels of IgG responses in pigs. Both formulations induced similarly high levels of IgG to p30 and p54 in pigs, which inferred that fusing OprI and TT-P2 to p30 and p54 has little effect on the immunogenicity of the fusion protein. This is most likely because p30 and p54 are highly immunogenic by nature [44]. In contrast, the inclusion of OprI and TT-P2 greatly enhanced the immunogenicity of the fusion proteins of p72 epitopes and △pE248R, and of △CD2v and △pEP153R (Fig. 4C-E). This finding suggests that the OprI fusion strategy together with TT-P2 is better able to enhance the immunogenicity of the fusion proteins consisting of less immunogenic ASFV proteins. Our results demonstrate that robust humoral immune responses are elicited by a coktail of OprI-fusion proteins formulated with ISA206, deepening our understanding of the functions of OprI and TT-P2 when fused to viral proteins.

Since it has been shown that cellular immune responses make a large contribution to protective immunity against intracellular infections with ASFV [22, 45–47], we determined proliferation of lymphocytes in PBMCs after in vitro stimulation. Pigs immunized with O-Ags-T formulation generated significantly higher level of lymphocyte proliferation than that of pigs immunized with Ags formulation, indicating that fusing OprI and TT-P2 to proteins/epitopes of ASFV notably enhanced cellular immune responses against ASFV. Furthermore, IFN-γ^+^ CD4^+^ and IFN-γ^+^ CD8^+^ T cells, which represent functional T lymphocytes, were also determined in our study. In accordance with lymphocyte proliferation, the percentages of IFN-γ^+^ CD4^+^ and IFN-γ^+^ CD8^+^ T cells in pigs immunized with O-Ags-T formulation were significantly higher than those of pigs immunized with Ags formulation or PBS. IFN-γ secreted by CD4^+^ and CD8^+^ T cells contributes to the polarization of the immune responses towards the Th1-type, and IFN-γ^+^ CD8^+^ T cells can exert cytolytic activity [48]. These results suggest that immunization with OprI-fused proteins cocktail favors activation of ASFV-specific T cells, may contribute to defend against ASFV infection.

Although higher levels of antigen-specific IgGs were detected in the sera from pigs vaccinated with O-Ags-T formulation, it remained unknown whether the immune sera were capable of neutralizing the virus. Therefore, neutralization assay were performed in vitro to analyze the ASFV neutralizing capability of the immune sera. The sera from pigs immunized with O-Ags-T formulation or Ags formulation neutralized more than 80% of ASFV in vitro. In earlier studies, the serum from pigs immunized with recombinant p30 expressed in baculovirus neutralized about 90% of ASFV, higher than those of our study [49]. The most likely reason for this discrepancy could be a higher amount of virus inoculation or different protein expression system employed in our study. In contrast, the ASFV neutralization percentage of the sera from p54 or p72 immunized pigs were relatively lower in the same study. More recently, the sera from pigs that received a combination of ASFV recombinant proteins and pcDNAs-expressing ASFV genes exhibited a similar ASFV neutralization percentage to ours in spite of different assays used in the neutralization test [50]. Interestingly, although pigs immunized with O-Ags-T formulation produced significant higher levels of IgG to p72, pE248R, CD2v and pEP153R than those of pigs immunized with Ags formulation, the virus was neutralized equally well by the sera from pigs that received either of them. This is likely because p30- and p54-specific IgGs play a dominant role in neutralization of ASFV, with no significant difference between two groups (Fig. 4A and 4B). We also found that ASFV was not completely neutralized by the sera from pigs immunized with either formulation, as shown in Fig. 7. Similar observations were reported in previous studies [51, 52], probably due to the complex nature of ASFV.

To investigate the effect of the cellular immunity on ASFV infection, virus reproduction in PBMCs from immunized pigs were determined. In accordance with the percentages of proliferative lymphocytes and IFN-γ^+^ CD8^+^ T cells, PBMCs from pigs immunized with O-Ags-T formulation or Ags formulation were shown to significantly inhibit ASFV infection in vitro relative to PBMCs in the control groups. Similar observations were reported in a previous study, where PBMCs from macaques vaccinated with live attenuated simian immunodeficiency virus (SIV) were significantly less sensitive to in vitro infection of virulent SIV than PBMCs from naive controls [53]. The virus inhibitory effects of PBMCs were attributed to virus-specific CD8^+^ T lymphocyte [54]. In line with this, PBMCs from pigs immunized with O-Ags-T formulation, showing significantly higher percentage of IFN-γ^+^ CD8^+^ T cells, inhibited nearly two fold higher percentage of ASFV infection in vitro than PBMCs from pigs immunized with Ags formulation. These data suggest that T lymphocytes activated by immunization with a cocktail of OprI-fused proteins are better able to inhibit ASFV infection in vitro, and may lead to efficient protective cellular immune responses against ASFV challenge.

The present work was undertaken as an extension of our previous work demonstrating that OprI and TT-P2 in OPMT contribute to enhance p30- and p54-specific immune responses in mice, and the antibody induced by OPMT showed certain neutralization activity against ASFV in vitro [55]. As a result, OprI and TT-P2 were used to construct OPET and OCET to improve the immunogenicity of p72 epitopes, pE248R, CD2v and pEP153R, which were shown to induce anti-ASFV immune responses [56]. Furthermore, humoral and cellular immune responses induced by a cocktail of OPMT, OPET and OCET in pigs, as well as the ASFV inhibitory effects of the immune sera and PBMCs, were evaluated in the present study. The findings in this study provides the basis for ASFV challenge experiments with O-Ags-T formulation.

## Conclusion

In summary, our work demonstrates that immunization with a cocktail of three recombinant OprI-fusion proteins, each containing 2 different ASFV proteins/epitopes fused with bacterial lipoprotein OprI and a universal CD4^+^ T cell epitope, formulated with ISA206 adjuvant induced potent ASFV-specific humoral and cellular immune responses in pigs. More importantly, the serum and PBMCs from the immunized pigs respectively inhibited 82.8% and 92.6% of ASFV infection in vitro, which is probably correlated with efficient protective immunity against ASFV challenge. Further studies should aim to evaluate the protective efficacy of O-Ags-T formulation as a vaccine candidate against lethal ASFV challenge in pigs, and it will be highly interesting to explore the role of different ASFV proteins in protective immune responses against ASFV. Overall, our study provides valuable information for the development of future subunit vaccines against ASF. 

## Supplementary Information

**Additional file 1**: **Fig. S1**. The gating strategy in the detection of IFN-γ-producing T cells in PBMCs from immunized pigs at 42 dpv.

**Additional file 2**: **Fig. S2**. The percentage of IFN-γ-producing CD4^+^ T cells (represented by P8) in PBMCs from each immunized pig at 42 dpv after in vitro stimulation with inactivated ASFV, mediumor a cell activation cocktail. Group 1 and group 2 respectively represent pigs immunized with O-Ags-T formulation or Ags formulation.

**Additional file 3**: **Fig. S3**. The percentage of IFN-γ-producing CD8^+^ T cells (represented by P5) in PBMCs from each immunized pig at 42 dpv after in vitro stimulation with inactivated ASFV, mediumor a cell activation cocktail. Group 1 and group 2 respectively represent pigs immunized with O-Ags-T formulation or Ags formulation.

## Data Availability

The datasets used and/or analyzed in this study are obtained and available from the corresponding authors upon a reasonable request.

## Abbreviations

ASF: African swine fever
ASFV: African swine fever virus
OprI: Major outer membrane lipoprotein I
TLR: Toll-like receptor
CD: Cluster of differentiation
BMDCs: Bone marrow-derived dendritic cells
PBMCs: Peripheral blood mononuclear cells
PAMs: Porcine alveolar macrophages
FBS: Fetal bovine serum
LVRI: Lanzhou Veterinary Research Institute
TT-P2: A universal tetanus toxoid CD4^+^ T cell epitope P2
CE: Truncated CD2v-truncated pEP153R
PE: p72 epitopes-truncated pE248R
PM: p30-modified p54
OCET: OprI-truncated CD2v-truncated pEP153R-TT
OPET: OprI-p72 epitopes-truncated pE248R-TT
OPMT: OprI-p30-modified p54-TT
OD: Optic density
EDTA: Ethylenediaminetetraacetic acid
LAL: Limulus amebocyte lysate
SDS-PAGE: Sodium dodecyl sulfate polyacrylamide gel electrophoresis
RT: Room temperature
mAb: Monoclonal antibody
PBS: Phosphate buffer saline
PBST: PBS containing 0.05% Tween-20
HRP: Horseradish peroxidase
LPS: Lipopolysaccharide
TNF-α: Tumor necrosis factor-α
IL-12p70: Interleukin-12p70
IFN-γ: Interferon-γ
O-Ags-T: A cocktail of OPMT, OPET and OCET
ELISA: Enzyme-linked immunosorbent assay
dpv: Days post vaccination
IgG: Immunoglobulin G
CFSE: 5(6)-Carboxyfluorescein diacetate succinimidyl ester
HAD_50_: 50% Hemadsorbing doses
AF647: Alexa Fluor 647
qPCR: Quantitative polymerase chain reaction
TRITC: Tetraethyl rhodamine isothiocyanate
MOI: Multiplicity of infection
SD: Standard deviation
NS: No significance
MHC: Major histocompatibility complex
