# Supplementary figures and images for: Evaluation of humoral and cellular immune responses induced by a cocktail of recombinant African swine fever virus antigens fused with OprI in domestic pigs
Source: Virol J. 2023 May 26;20:104. doi: 10.1186/s12985-023-02070-7 (PMC10224232; doi:10.1186/s12985-023-02070-7)

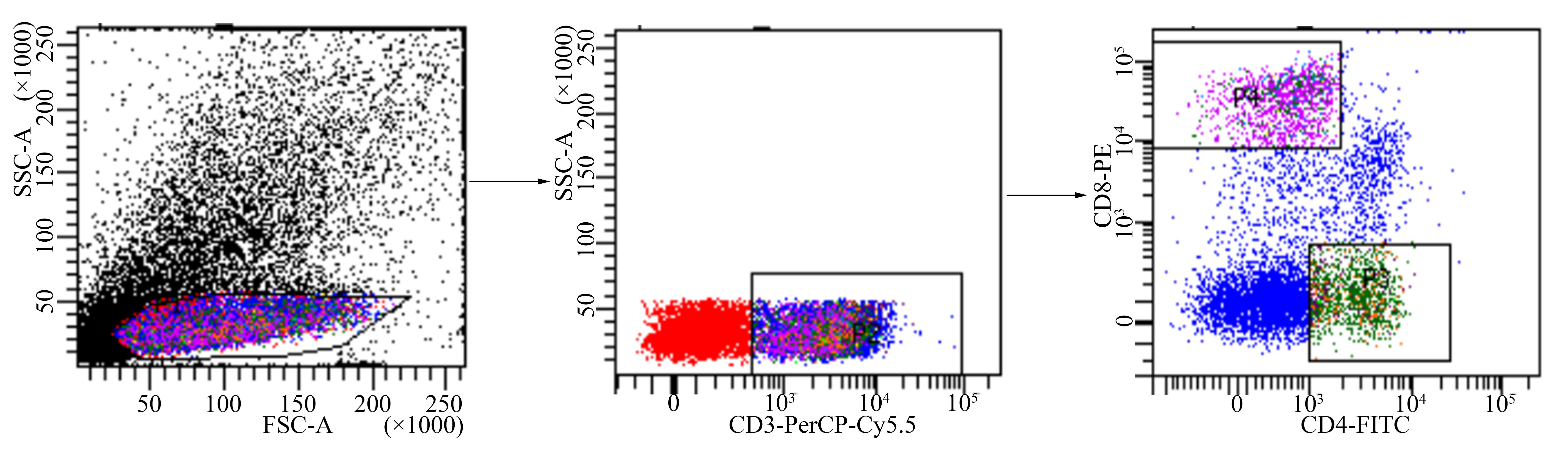

Supplement: Supplementary file 1 — Additional file 1: Fig. S1. The gating strategy in the detection of IFN-γ-producing T cells in PBMCs from immunized pigs at 42 dpv. [file 12985_2023_2070_MOESM1_ESM.tif]

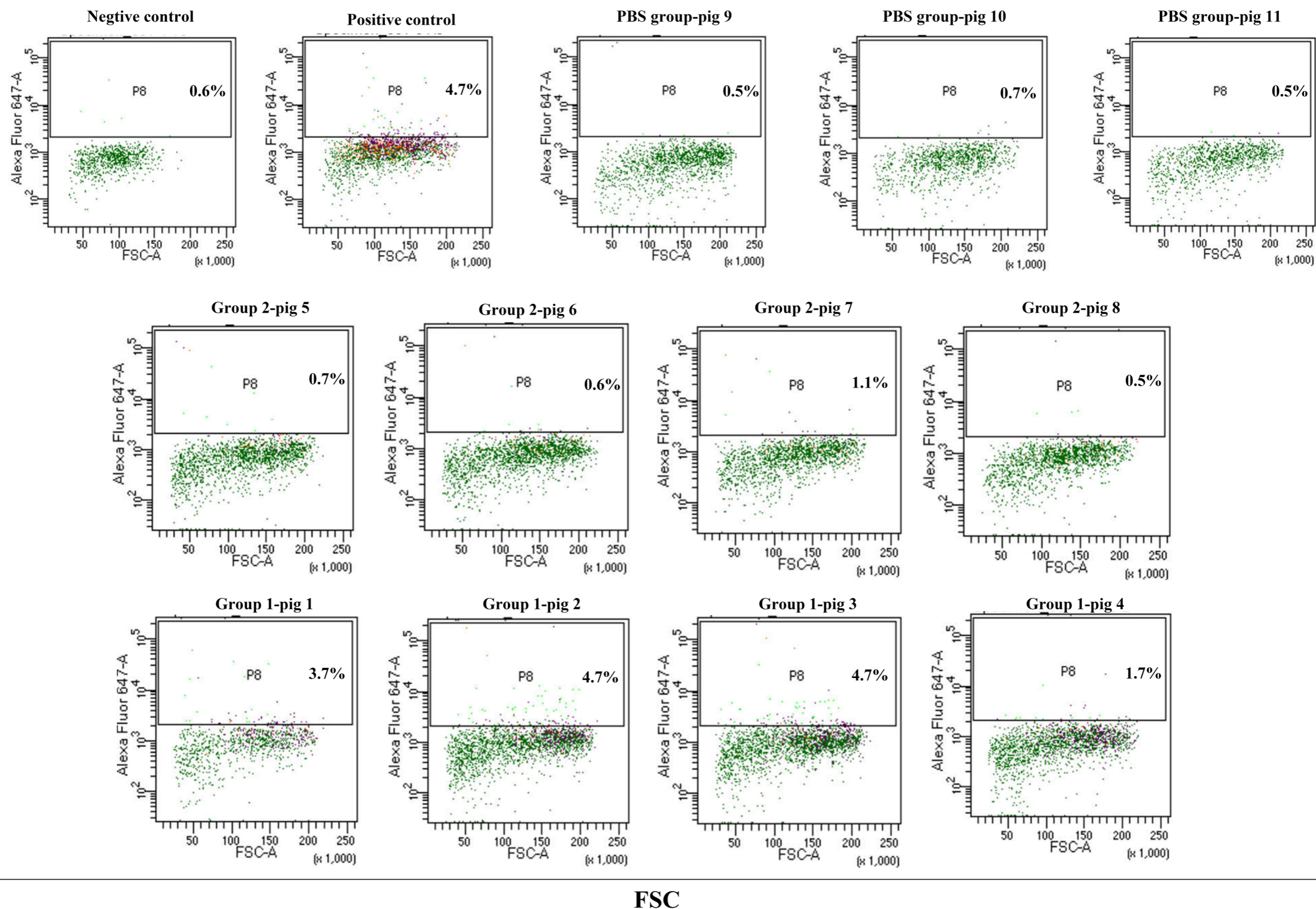

Supplement: Supplementary file 2 — Additional file 2: Fig. S2. The percentage of IFN-γ-producing CD4+ T cells (represented by P8) in PBMCs from each immunized pig at 42 dpv after in vitro stimulation with inactivated ASFV, mediumor a cell activation cocktail. Group 1 and group 2 respectively represent pigs immunized with O-Ags-T formulation or Ags formulation. [file 12985_2023_2070_MOESM2_ESM.pdf]

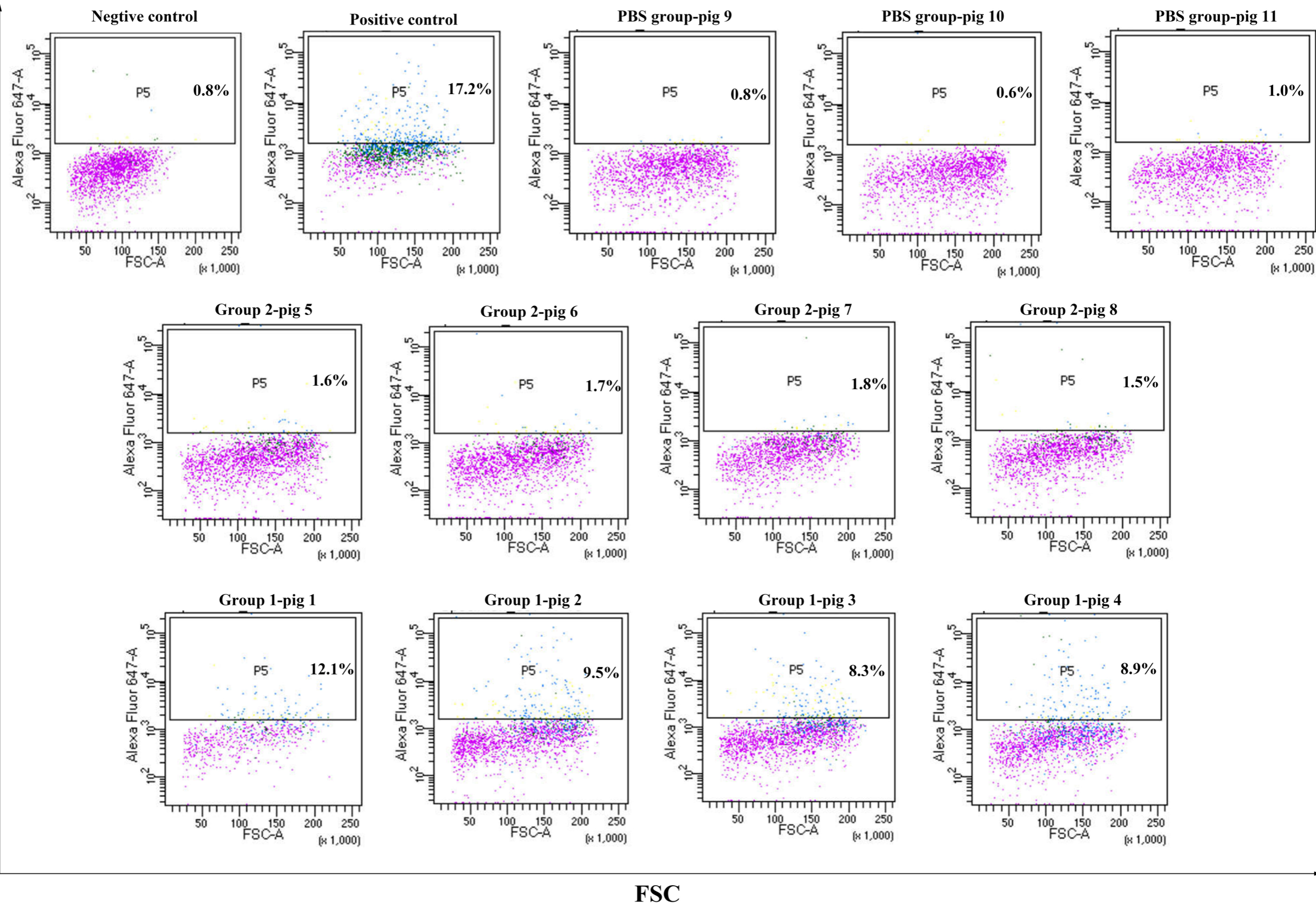

Supplement: Supplementary file 3 — Additional file 3: Fig. S3. The percentage of IFN-γ-producing CD8+ T cells (represented by P5) in PBMCs from each immunized pig at 42 dpv after in vitro stimulation with inactivated ASFV, mediumor a cell activation cocktail. Group 1 and group 2 respectively represent pigs immunized with O-Ags-T formulation or Ags formulation. [file 12985_2023_2070_MOESM3_ESM.pdf]
